# Generalized ComBat harmonization methods for radiomic features with multi-modal distributions and multiple batch effects

**DOI:** 10.1038/s41598-022-08412-9

**Published:** 2022-03-16

**Authors:** Hannah Horng, Apurva Singh, Bardia Yousefi, Eric A. Cohen, Babak Haghighi, Sharyn Katz, Peter B. Noël, Russell T. Shinohara, Despina Kontos

**Affiliations:** 1 Department of Bioengineering, University of Pennsylvania, Philadelphia, PA 19104 USA; 2 Department of Radiology, Center for Biomedical Image Computing and Analysis (CBICA), University of Pennsylvania, Philadelphia, PA 19104 USA; 3 Laboratory for Advanced Computed Tomography Imaging, Department of Radiology, University of Pennsylvania, Philadelphia, PA 19104 USA; 4 Penn Statistics in Imaging and Visualization Endeavor (PennSIVE), Department of Biostatistics, Epidemiology and Informatics, University of Pennsylvania, Philadelphia, PA 19104 USA

**Keywords:** Prognostic markers, Computed tomography, Statistics

## Abstract

Radiomic features have a wide range of clinical applications, but variability due to image acquisition factors can affect their performance. The harmonization tool ComBat is a promising solution but is limited by inability to harmonize multimodal distributions, unknown imaging parameters, and multiple imaging parameters. In this study, we propose two methods for addressing these limitations. We propose a sequential method that allows for harmonization of radiomic features by multiple imaging parameters (Nested ComBat). We also employ a Gaussian Mixture Model (GMM)-based method (GMM ComBat) where scans are split into groupings based on the shape of the distribution used for harmonization as a batch effect and subsequent harmonization by a known imaging parameter. These two methods were evaluated on features extracted with CapTK and PyRadiomics from two public lung computed tomography datasets. We found that Nested ComBat exhibited similar performance to standard ComBat in reducing the percentage of features with statistically significant differences in distribution attributable to imaging parameters. GMM ComBat improved harmonization performance over standard ComBat (− 11%, − 10% for Lung3/CAPTK, Lung3/PyRadiomics harmonizing by kernel resolution). Features harmonized with a variant of the Nested method and the GMM split method demonstrated similar c-statistics and Kaplan–Meier curves when used in survival analyses.

## Introduction

In recent years, radiomics, or the extraction of quantitative features from imaging data, has emerged as a major field of study for a wide range of applications in oncology and precision medicine^1^. Multicenter studies are a necessity for radiomics to enable analyses with greater statistical power and generalizability, but imaging protocols often vary by institution in acquisition protocols, image post-processing, and reconstruction. The resulting heterogeneous datasets are broadly equivalent clinically but can often have differences that, although clinically subtle, can affect radiomic feature extraction and analysis^2^. For example, recent studies in computed tomography (CT) of the lung have shown that reconstruction kernel and slice thickness can affect the radiomic features as well as the subsequent analyses to find homogenous lung disease subgroups and assess lung texture patterns^3,4^. The problem is not unique to CT, as magnetic resonance (MR) imaging intensity is also highly dependent on manufacturer, sequence, and acquisition parameters^5^. A recent study of cervix MR showed that few MR features were robust across scanners and acquisition parameters, while another study of brain MR demonstrated that MR-derived radiomic features vary widely with pulse sequence^6,7^.

Many standardization approaches have been developed to address this problem, which can be broadly grouped into the image domain and the feature domain. Approaches in the image domain attempt to correct for differences in acquisition and reconstruction prior to feature extraction, including the following: standardizing protocols, incorporating robustness into feature definitions, and image preprocessing^8^. However, these procedures are often not implemented or require modification of existing guidelines for standardized radiomic feature extraction. Approaches in the feature domain correct unwanted variation after feature extraction, including selecting for robust features and batch effect correction methods^8^. Feature selection can eliminate features with unwanted variation due to technical factors and help alleviate collinearity, but also can result in the loss of information that could otherwise be useful in further analysis. Batch effect correction methods enable standardization following extraction with existing open-source tools without further loss of information, where batch effects are non-biological factors that alter resulting data^8^.

One such batch effect correction method is ComBat, a harmonization method originally developed for genomics that can address and correct variation in imaging features due to imaging parameters by using empirical Bayes to estimate location and scale parameters^9,10^. In previous studies, ComBat has been shown to harmonize radiomic features from different CT protocols as well as reduce the number of features with significantly different distributions by batch effect^11,12^. While ComBat is fast and easy to use, it also has several limitations. The first is that the method assumes that errors from the standardized input data will follow a normal distribution, which may not always be the case feature distributions can appear multimodal. The second is that ComBat assumes that all batch effects and clinical covariates are known, and therefore cannot correct or preserve variation due to any factors not included in the dataset. Finally, while datasets are often heterogeneous in more than one batch effect, current implementations of ComBat are only able to harmonize by a single batch effect at a time.

In this work, we propose two methods of addressing the above limitations to improve ComBat performance in harmonizing radiomic features. In the Nested approach, radiomic features are sequentially harmonized to handle multiple batch effects in datasets heterogeneous in more than one imaging parameter. In the Gaussian Mixture Model (GMM) approach, scan groupings are automatically identified and used to remove variation due to unknown covariates as well as transform bimodal data into Gaussian components in datasets with bimodal feature distributions attributable to unknown batch effects. These generalizations of the ComBat method promise improved harmonization in the context of increasingly popular radiomic approaches with multiple, complex batch effects. We then demonstrate their application on publicly available lung CT images to remove variation due to reconstruction kernel, manufacturer, and the use of intravenous contrast.

## Results

### Nested ComBat

**Figure 1:**
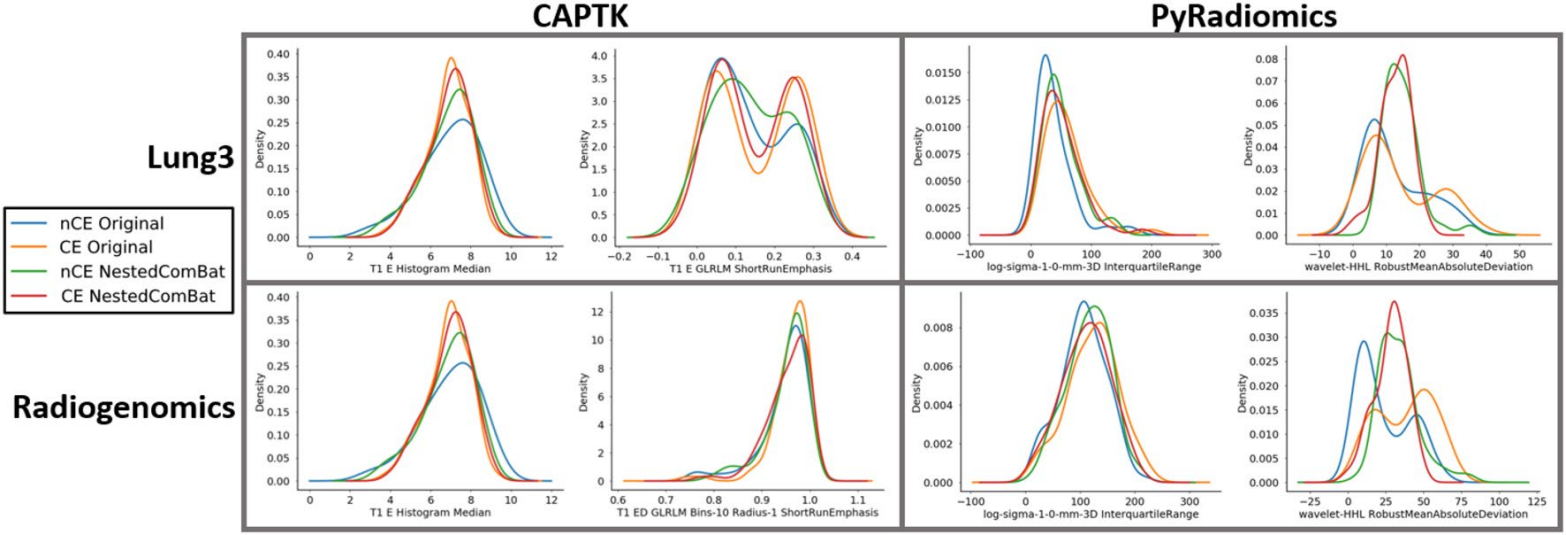
Representative kernel density plots for the original features and after applying Nested ComBat. Kernel density plots represent Nested ComBat results split on contrast enhancement, where nCE indicates no enhancement and CE indicates enhancement. Harmonization should result in more similar feature distributions. Within each combination of feature extraction package and software, the plot on the left illustrates the effect of Nested ComBat on a Gaussian distribution, while the plot on the right illustrates the effect of Nested ComBat on a bimodal distribution.

The results of both Nested ComBat and Nested Dropped (NestedD) ComBat are shown in Table 1 and Fig. 1. It was visually observed that while Nested ComBat harmonized some of the distributions by making the kernel density plots more similar, it was not as effective when feature distributions were bimodal in shape, a characteristic shown in the histograms for ShortRunEmphasis (Fig. 1). For NestedD ComBat, 14% and 24% of features were dropped in the Lung3 dataset for CapTK and PyRadiomics, respectively. These features were only dropped in the NestedD approach, and these percentages are not equivalent to the percentage of features with significantly different distributions attributable to batch effects in the original data and post-harmonization. In the Radiogenomics dataset, 28% and 27% of features were dropped for the CapTK and PyRadiomics datasets, respectively (Table 1). Nested ComBat exhibited similar performance to the standard ComBat implementation in reducing the number of features with significant differences in distribution due to batch effect in both the Radiogenomics and Lung3 datasets for both the CapTK and PyRadiomics features (+ 2%, + 4%, − 7%, − 6% for Lung3/CAPTK, Lung3/PyRadiomics, Radiogenomics/CAPTK, Radiogenomics/PyRadiomics when harmonizing by spatial resolution), and in some cases increased the percentage of features with significant differences in distribution due to a batch effect. However, applying NestedD ComBat resulted in fewer features with significant differences in all radiomic feature sets when compared to standard and Nested ComBat, as measured by the percentage out of the original number of features with detected significant (*p* < 0.05) differences in distribution (− 2%, − 14%, − 32%, − 16%, for Lung3/PyRadiomics, Radiogenomics/CAPTK, Radiogenomics/PyRadiomics when harmonizing by spatial resolution comparing standard vs. NestedD ComBat). In addition, there was a greater proportion of features with significant differences before ComBat with PyRadiomics features than with CapTK features for both the Lung3 and Radiogenomics datasets.

**Table 1:** (A) Percentage of features with significantly different distributions attributable to contrast enhancement, spatial resolution due to reconstruction kernel, and manufacturer in the original features and after applying standard ComBat, Nested ComBat, and NestedD (dropping with every iteration) ComBat in the CapTK features extracted from the Lung3 dataset. (B) Corresponding table for PyRadiomics features extracted from the Lung3 data. (C) Corresponding table for CapTK features extracted from the Radiogenomics dataset. (D) Corresponding table for PyRadiomics features extracted from the Radiogenomics dataset.

	Original (%)	ComBat (%)	Nested (%)	NestedD (%)
**A. Lung3/CAPTK**				
CE	10	16	5	3
Spatial resolution	18	21	23	19
Manufacturer	48	45	41	28
**B. Lung3/PyRadiomics**				
CE	40	11	5	2
Spatial resolution	43	25	29	11
Manufacturer	61	28	27	12
**C. Radiogenomics/CAPTK**				
CE	17	42	33	14
Spatial resolution	42	43	36	11
Manufacturer	20	51	38	15
**D. Radiogenomics/PyRadiomics**				
CE	54	27	26	9
Spatial resolution	69	29	23	13
Manufacturer	44	36	44	20

Tables contain the percentage of features out of the original number of features with detected significant (*p* < 0.05) differences in distribution for all batch effects.

### Gaussian mixture model (GMM) ComBat

**Figure 2:**
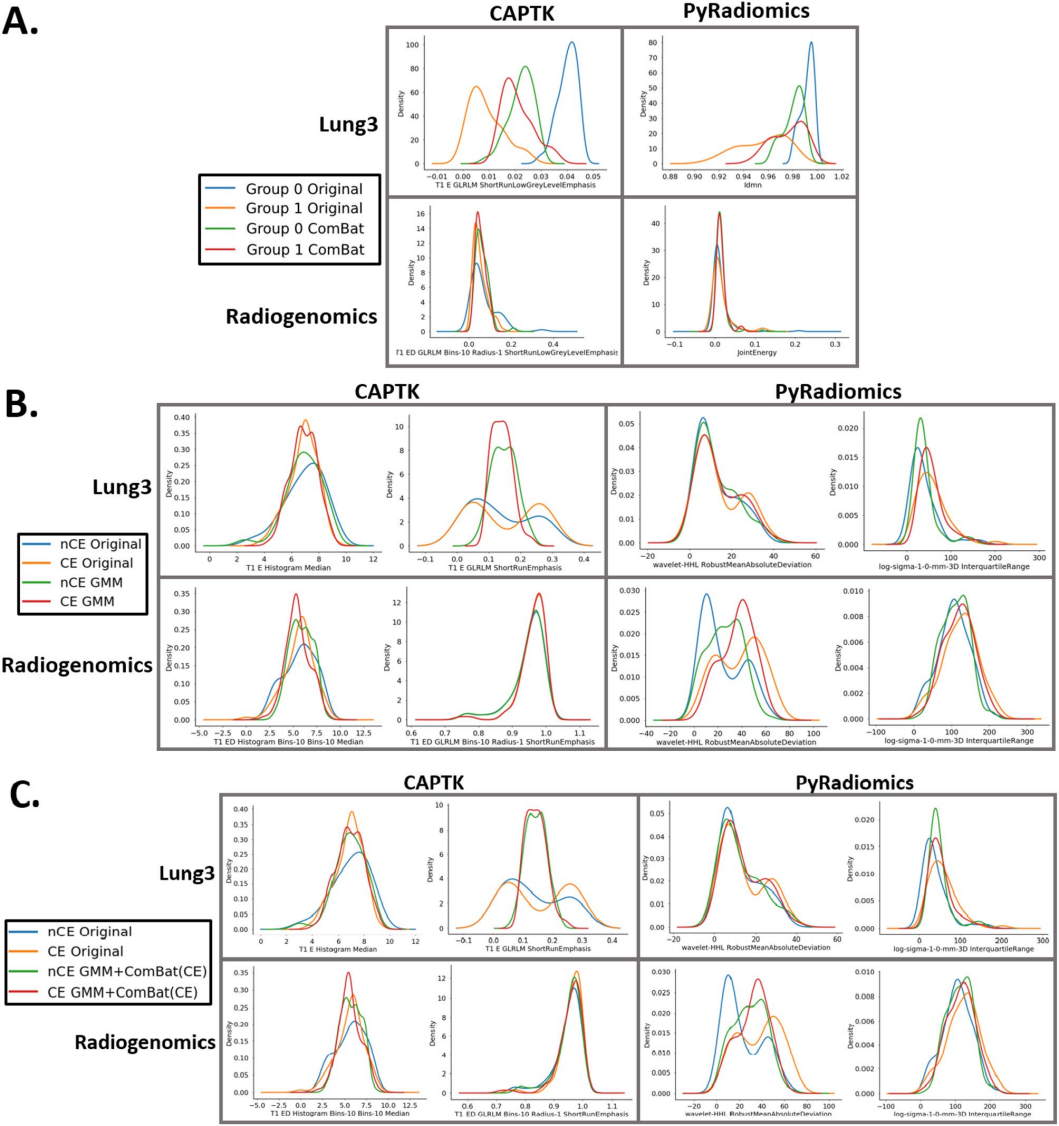
(**A**) Kernel density plots for the feature used to generate the GMM grouping before and after harmonization by the GMM groupings. B) Representative kernel density plots for the original features and after applying standard ComBat and harmonizing by the GMM grouping alone (GMM). C) Representative kernel density plots for the original features and after harmonizing by both the GMM grouping and known imaging parameter batch effects (GMM + ComBat (CE)). Kernel density plots represent ComBat results separated by the batch variable contrast enhancement, where nCE indicates no enhancement and CE indicates enhancement. For (**B**) and (**C**), representative features whose distributions best visually demonstrate the effects of GMM ComBat were selected by screening all the feature distributions before and after harmonization. Harmonization should result in more similar feature distributions.

The results of harmonizing by the scan grouping generated with a GMM are shown in Table 2 and Fig. 2. Applying ComBat to harmonize by the GMM grouping reduces the percentage of features significantly different in their distributions due to the unknown batch effect inferred from the GMM grouping (− 43%, − 58%, − 28%, − 45% for Lung3/CapTK, Lung3/PyRadiomics, Radiogenomics/CapTK, and Radiogenomics/PyRadiomics when harmonizing by GMM grouping) (Table 2A). Harmonizing by the GMM grouping alone did not decrease the percentage of features with significant differences in distributions attributable to the known imaging parameters in both datasets and in many cases failed to outperform standard ComBat (+ 7%, + 19%, + 2%, + 33% for Lung3/CapTK, Lung3/PyRadiomics, Radiogenomics/CapTK, and Radiogenomics/PyRadiomics, respectively, when harmonizing by spatial resolution) (Table 2B). Subsequent harmonization by known imaging parameters reduced the percentage of features with significant differences in distribution due to the corresponding parameter when compared to harmonizing by the GMM grouping alone (− 18%, − 29%, − 20%, − 43% for Lung3/CapTK, Lung3/PyRadiomics, Radiogenomics/CapTK, and Radiogenomics/PyRadiomics, respectively, when harmonizing by spatial resolution) (Table 2B).

**Table 2:** (A) Percentage of features with significant differences in distribution before and after harmonization by the GMM groupings. Feature names indicate the feature whose distribution was used to generate the GMM scan grouping. GMM scan groupings are obtained by selecting the best GMM model from a set composed of GMM models generated from each of the features such that the final GMM scan grouping is estimated from a single feature. (B) Percentage of features with significantly different distributions attributable to batch effects in the original features and after applying standard ComBat, harmonizing by the GMM grouping alone (GMM), and harmonizing by both the GMM grouping and known imaging parameter batch effects (GMM + ComBat (CE)).

A	Original (%)	ComBat (%)		
**Lung3/CAPTK**				
T1_E_GLRLM_Short RunLowGreyLevel emphasis	88	45		
**Lung3/PyRadiomics**				
Idmn	84	26		
**Radiogenomics/CAPTK**				
T1_ED_GRLRLM_Bins-10_Radius-1_ShortRun LowGreyLevelEmphasis	78	50		
**Radiogenomics/PyRadiomics**				
Jointenergy	75	30		

B	Original (%)	ComBat (%)	GMM (%)	GMM + ComBat (%)
**Lung3/CAPTK**				
CE	10	16	4	4
Spatial resolution	18	21	28	10
Manufacturer	48	45	7	4
**Lung3/PyRadiomics**				
CE	40	11	35	7
Spatial resolution	43	25	44	15
Manufacturer	61	28	43	23
**Radiogenomics/CAPTK**				
CE	17	42	18	12
Spatial resolution	42	43	45	25
Manufacturer	20	51	17	25
**Radiogenomics/PyRadiomics**				
CE	54	27	47	16
Spatial resolution	69	29	62	19
Manufacturer	44	36	40	23

Tables contain the percentage of features out of the original number of features with detected significant (*p* < 0.05) differences in distribution for all batch effects.

### Method evaluation

**Figure 3:**
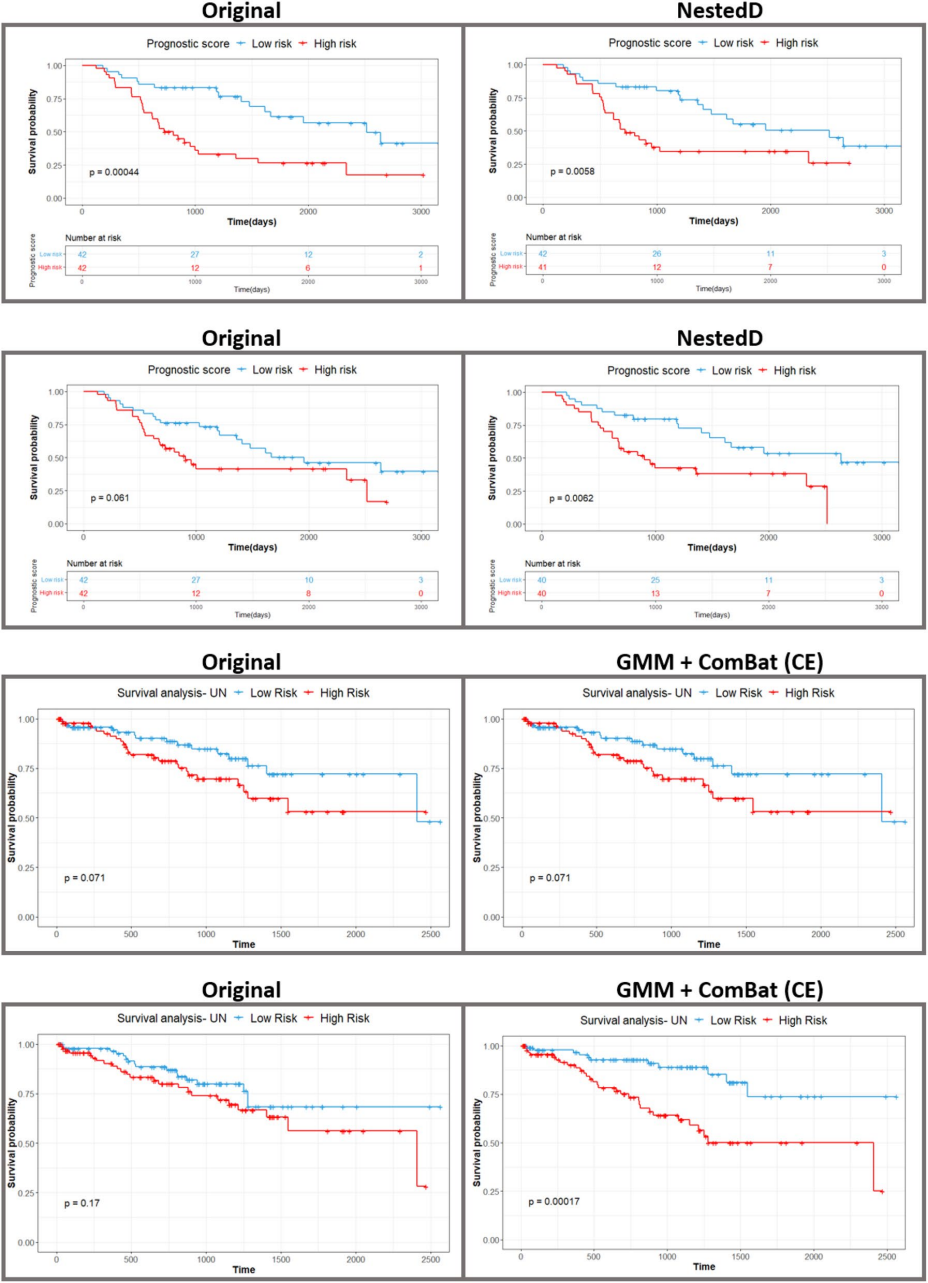
Survival analysis. In-sample Kaplan–Meier curves fitted on the original features and the harmonization approach with the highest c-statistic for each dataset.

The results of survival analyses completed with the original versus harmonized features are shown in Table 3 and Fig. 3. The NestedD harmonization approach yielded the highest fivefold cross-validated c-statistic (0.63 for CapTK, 0.64 for PyRadiomics) for the Lung3 dataset, an improvement over the c-statistics for models built on the original feature data (0.59 for CapTK, 0.62 for PyRadiomics). The original features and features harmonized with NestedD showed similar log-rank test *p*-values for the Kaplan–Meier curves: 0.0004 and 0.0058 for CapTK and 0.061 and 0.0062 for PyRadiomics. Using the standard ComBat implementation to harmonize by contrast enhancement resulted in models with c-statistics lower than models built with NestedD features (0.60 for CapTK, 0.61 for PyRadiomics). Standard ComBat resulted in a log-rank test *p*-value of 0.0029 for CapTK features and a corresponding value of 0.029 in PyRadiomics features. In contrast, the GMM + ComBat (CE) and ComBat (CE) methods had the highest c-statistic (0.58 for CapTK, 0.64 for PyRadiomics) for the Radiogenomics dataset, still greater than the c-statistics for models built on the original features data (0.55 for CapTK, 0.57 for PyRadiomics). Using the standard ComBat implementation to harmonize by contrast enhancement resulted in a log-rank test *p*-value of 0.056 for CapTK features and 0.0003 in PyRadiomics features. In addition, survival analyses were completed for the original, Nested-harmonized, and GMM-harmonized features in which features with a statistically significant difference in distribution observed with at least one imaging parameter were removed from the dataset (DROP) (Table S1, Fig. S1). In the Lung3 dataset, the Nested + DROP approach did not improve the c-statistic (0.63 for CapTK, 0.64 for PyRadiomics) over the NestedD approach. In the Radiogenomics dataset, the Nested + DROP approach showed an increased c-statistic (0.63 for CapTK, 0.65 for PyRadiomics) when compared to the GMM + ComBat (CE) approach. However, c-statistics from the different approaches were observed to be similar, as indicated by the 95% CI.

**Table 3:** C-statistics and 95% confidence intervals (CI) for fivefold cross-validated Cox proportional hazard models built from harmonized data, and log-rank *p*-values for Kaplan–Meier curve separation. ComBat (CE) indicates data was harmonized by contrast enhancement with ComBat.

	5fold CV c-statistic	95% CI	Log-rank *p*-value
**Lung3/CAPTK**			
Original	0.59	[0.53, 0.65]	0.0004
ComBat (CE)	0.60	[0.54, 0.64]	0.0029
Nested	0.63	[0.59, 0.67]	0.0025
NestedD	0.63	[0.58, 0.68]	0.0058
GMM	0.50	[0.42, 0.56]	0.011
GMM + ComBat (CE)	0.50	[0.42, 0.56]	0.036
**Lung3/PyRadiomics**			
Original	0.62	[0.57, 0.66]	0.061
ComBat (CE)	0.61	[0.57, 0.66]	0.029
Nested	0.62	[0.56, 0.67]	0.022
NestedD	0.64	[0.58, 0.69]	0.0062
GMM	0.59	[0.53, 0.65]	0.01
GMM + ComBat (CE)	0.58	[0.52, 0.63]	0.016
**Radiogenomics/CAPTK**			
Original	0.55	[0.52,0.63]	0.071
ComBat (CE)	0.55	[0.50,0.59]	0.056
Nested	0.56	[0.52,0.63]	0.016
NestedD	0.54	[0.53,0.65]	0.074
GMM	0.56	[0.51,0.64]	0.02
GMM + ComBat (CE)	0.58	[0.53,0.64]	0.071
**Radiogenomics/PyRadiomics**			
Original	0.57	[0.52,0.63]	0.17
ComBat (CE)	0.63	[0.53,0.67]	0.0003
Nested	0.62	[0.53,0.69]	0.004
NestedD	0.61	[0.53,0.68]	0.0078
GMM	0.61	[0.51,0.66]	0.0012
GMM + ComBat (CE)	0.63	[0.52,0.68]	0.0002

## Discussion

In this work, we first propose Nested ComBat to enable ComBat harmonization by multiple batch effects in datasets heterogeneous in multiple imaging parameters. We then develop GMM ComBat to enable ComBat harmonization by bimodal distributions, where the bimodality is assumed to be caused by an unknown imaging parameter. We found that Nested ComBat exhibited similar harmonization performance to standard ComBat in reducing the number of features with statistically significant differences in distribution attributable to batch effect, likely due to the presence of bimodal feature distributions. GMM ComBat, a harmonization method designed to handle bimodal distributions, improved harmonization performance over standard ComBat. Features harmonized with these new approaches demonstrated similar c-statistics and Kaplan–Meier curves when used in survival analysis.

Imaging datasets are often heterogeneous in more than one imaging parameter (the Radiogenomics and Lung3 datasets varied in manufacturer and contrast enhancement, as well as spatial resolution due to reconstruction kernel). The standard ComBat implementation is only capable of harmonizing by a single batch effect at a time, necessitating the development of Nested ComBat to sequentially harmonize by each batch effect, when multiple batch effects may be present. However, applying Nested ComBat did not reduce the percentage of features significantly different in their distribution across the imaging parameters harmonized (Table 1). This is likely because several of the features have a distribution that is bimodal in shape as opposed to Gaussian (Fig. 1). ComBat relies on several statistical assumptions to estimate the parameters used to shift and scale the data. Bimodal distributions violate these assumptions, resulting in poor performance in harmonizing bimodal data. One potential solution is the NestedD algorithm in which all the features with significant differences in distribution were dropped at every iteration, essentially dropping all features who retain bimodality after each nested harmonization step. While this improved performance in reducing the number of features with significantly different distributions by batch effect, the process of dropping features results in loss of information that should ideally be preserved by using ComBat harmonization.

In certain instances, the bimodality may be due to a variable not measured in the study, which can be expected given that image datasets will not always come with a sufficiently extensive list of clinical covariates and imaging parameters; indeed, in many cases unwanted variability may be due to unknown factors. In many cases these factors may even be unknown to the clinicians and technicians responsible for compiling the dataset. For example, a clinical variable like body mass index (BMI) could affect image quality and cause bimodality in the feature distributions but could also not included in the dataset, making the cause of the bimodality unknown to the researcher. The GMM split method is an approach to solving this problem by assuming that although the variable causing the bimodal shape is unknown, the scan groupings for this hidden variable can be estimated from the distribution of an imaging feature itself. Groupings generated from the GMM split method do not improve performance when the features are harmonized by the grouping alone but do substantially reduce the percentage of features with significant differences in distribution due to batch effects when subsequently harmonized by those batch effects (Table 2). However, it was visually observed that the two distributions generated from the GMM model are Gaussian in shape and increase in overlap following harmonization (Fig. 2A). Some of the features that appeared bimodal in Nested ComBat were no longer bimodal following harmonization with the GMM grouping and known batch effects (Fig. 2B). The GMM method for selecting the scan grouping is fully automated and requires no manual review (as the best model is selected using the AIC) and can take less time to run than visually generating the split, while generating more reproducible results.

However, this method is not without their limitations. Ideally the variable causing the bimodality should be known, but because the scan groupings for the hidden variable are estimated from a single feature distribution, the grouping does not necessarily split all feature distributions into Gaussian components. Thus, some features remain bimodal even after applying harmonization with the split method (Fig. 2B). In addition, the hidden variable could be strongly associated with a clinical covariate of interest that could contain useful information for further analyses. In this work, we assume that all clinical covariates are known and protected during harmonization. While the GMM split method can be used to handle bimodality in radiomic feature datasets given the standard ComBat implementation, future work could improve the statistical methodology behind ComBat to better handle non-Gaussian or bimodal distributions. Another potential modification is modeling a separate GMM for each feature to generate a unique scan grouping per feature, which would address the lack of generalizability when applying a scan grouping from one to all features. However, because the scan grouping for each feature would be different, this approach would require separate harmonization for each feature.

Results of the survival analysis show that using data harmonized with modified ComBat can improve the model quality of subsequent analyses, as shown by the increased the c-statistics and separations between Kaplan–Meier curves. However, which approach most consistently produces the best model is unclear NestedD showed better performance for the Lung3 dataset, while the split methods had better performance for the Radiogenomics dataset. Dropping features with statistically significant differences in distribution following harmonization also demonstrated inconsistent performance, as the DROP approaches improved the c-statistics in the Radiogenomics dataset but not the Lung3 dataset. The DROP approaches are not ideal given that the dropping of features could result in loss of information useful to predictive analyses. Future work, with larger datasets, could include determining if combining nested and split harmonization approaches improves performance over using either alone. In addition, standard ComBat (CE) performed comparably to split methods in the Radiogenomics PyRadiomics feature set despite having a greater proportion of features with significantly different distributions. This shows that having reduced percentage of features with significantly different distributions is not guaranteed to improve performance in subsequent analysis. One potential reason for these results is that harmonizing by the split groupings could eliminate a factor that would otherwise improve model predictive power (i.e. eliminating an unknown clinical covariate). Another is that having significantly different distributions when split by a batch effect via the KS test is not necessarily indicative of a feature being affected by unwanted variation due to an imaging parameter, implying a need for better statistical testing methods for detecting features with such unwanted variation.

In the original features, it was observed that there was a greater proportion of features with significant differences with PyRadiomics features than with CapTK features for both the Lung3 and Radiogenomics datasets, possibly because CapTK is standardized per the International Biomarker Standardization Initiative (IBSI) criteria. While PyRadiomics is for the most part compliant with IBSI criteria, there are some differences in gray value discretization and binning that may be contributing to the increased proportion of features with significant differences due to batch effects.

**Figure 4:**
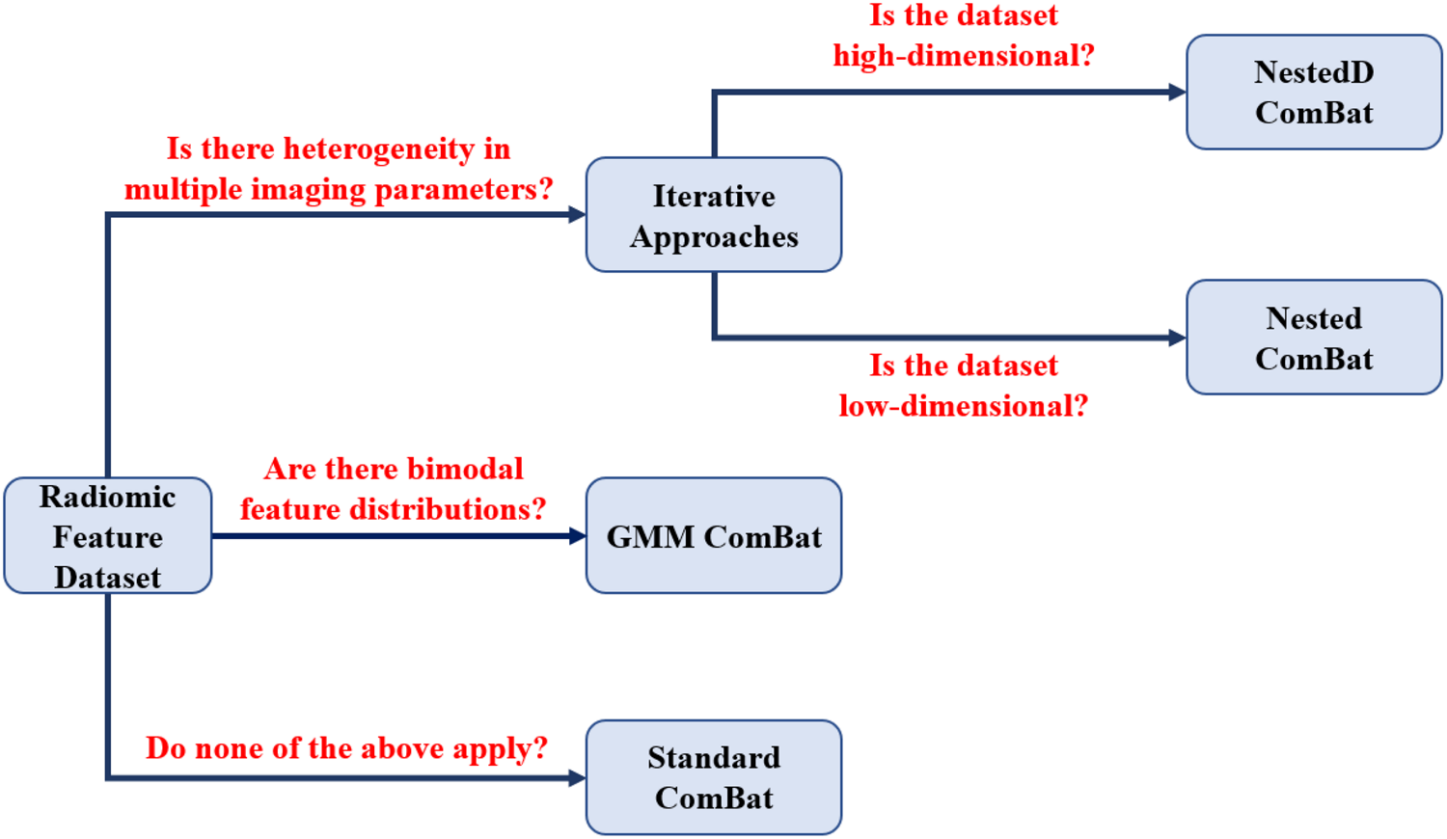
Decision flowchart indicating the context most suitable for each of the evaluated approaches.

Each method evaluated in this work was developed for a specific context. Nested ComBat and NestedD ComBat were both designed for datasets heterogeneous in multiple imaging parameters. NestedD ComBat is more suitable for higher dimensional datasets, where the effects of loss of information resulting from the dropping of features is reduced. GMM ComBat and its variants are designed for multimodal feature distributions where the multimodality is caused by some unknown imaging parameter or clinical variable. These recommendations are summarized in Fig. 4.

In this work, we have developed nested and split algorithms for ComBat harmonization that can better reduce the number of radiomic features with significantly different distributions attributable to imaging factors by addressing the limitations of the original ComBat implementation. We have shown that radiomic features harmonized with these approaches can yield better performance in further analyses, as demonstrated by the results of the survival analysis, as well as potentially improving study reproducibility. Studies with additional, larger, datasets (particularly with other modalities besides CT) are needed to further validate our findings.

## Material and methods

### Statistical testing

The Kolmogorov–Smirnov (KS) test was used to assess for general differences between feature distributions. This test was favored over the Wilcoxon-Rank Sum test given that some observed distributions appeared multimodal. The percentage of features out of the original number of features with detected significant (*p* < 0.05) differences in distribution due to an individual batch effect was used as a metric measuring the success of ComBat in eliminating variation caused by the corresponding batch effect. For methods that involved dropping features, the percentage was reported as out of the original number of features as opposed to the number of remaining features.

### Nested ComBat

**Figure 5:**
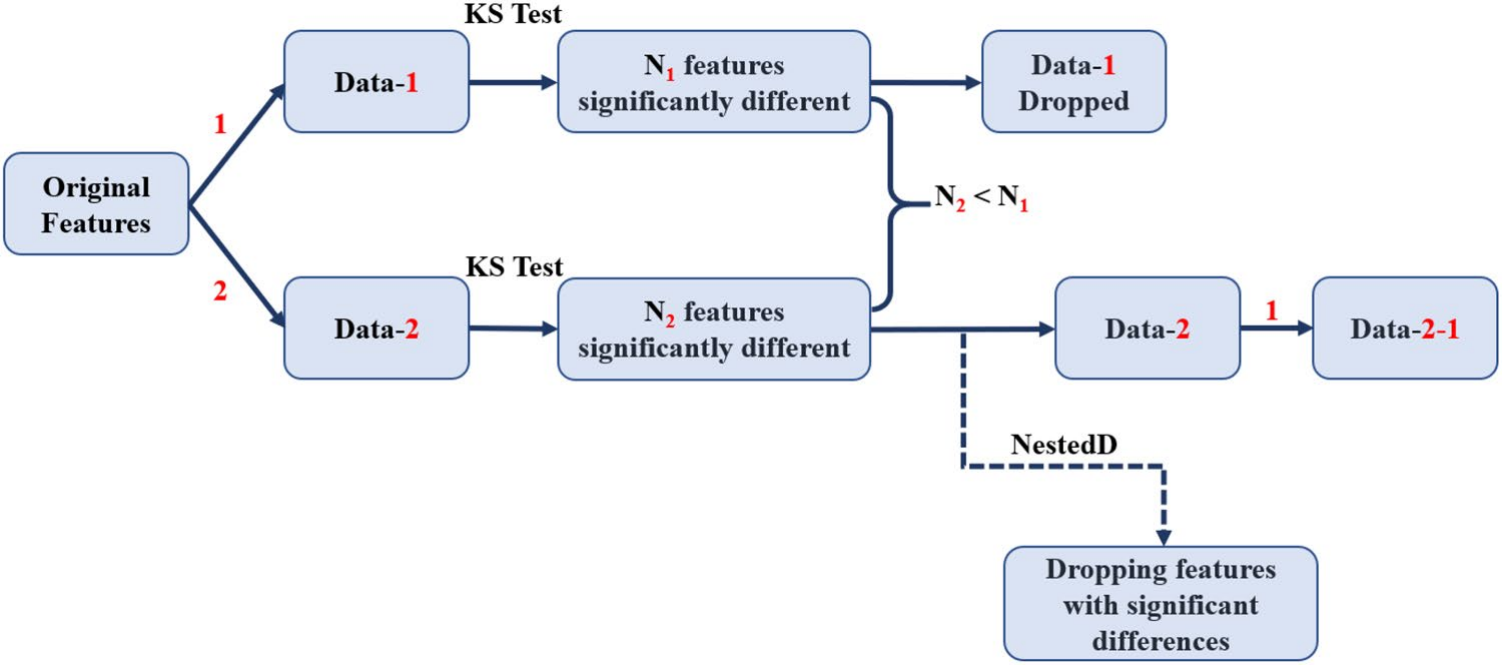
Workflow for the Nested ComBat implementation for sequential harmonization given two batch effects. Red denotes a batch effect, while the dash indicates that the data has been harmonized by a particular batch effect (i.e., Data-1 means the data has been harmonized by batch effect 1).

Given that the original implementation of ComBat only harmonizes by a single batch effect at a time, we designed a procedure for sequentially harmonizing by multiple batch effects called Nested ComBat (Fig. 5). The process is initialized with a list of batch effects and the original radiomic features as the input data. At each iteration, the features were first separately harmonized by each batch effect in the list, with variation due to clinical variables protected. The batch effects that had been used for harmonization in earlier iterations were not protected, as it was assumed that any variation due to these batch effects was undesirable. The resulting harmonized feature sets were each assessed for significant differences between distribution due to individual batch effects with the KS test. The harmonized feature set with the lowest number of features with detected differences in distribution was selected as the input for the next iteration, with the corresponding batch effect removed from the list. The procedure was repeated until there were no batch effects remaining in the list, with a single feature set sequentially harmonized by all n batch effects returned. The output feature set was assessed for significant differences in distribution when split by each of the original batch effects.

In addition to this Nested ComBat (Nested), we created a variation where features with significant differences in distribution were removed from the dataset after each iteration (NestedD) to evaluate the hypothesis that selecting for more robust features during sequential harmonization could result in greater harmonization performance. In addition, further evaluation was needed to determine if the loss of information associated with removing features from the dataset in this approach affected the downstream predictive analyses.

The code for implementing all algorithms developed in this work can be found at https://github.com/hannah-horng/generalized-combat.

### Gaussian mixture model (GMM) ComBat

**Figure 6:**
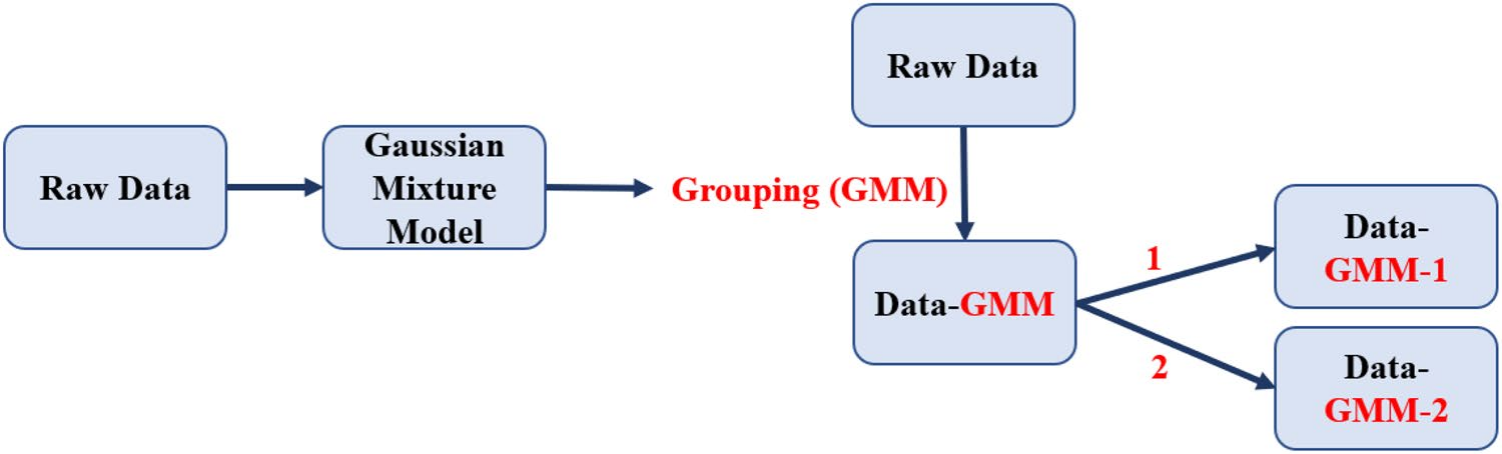
Workflow for the Gaussian mixture model (GMM) ComBat implementations. Red denotes a batch effect, while the dash indicates that the data has been harmonized by a particular batch effect (i.e., Data-1 indicates that the data has been harmonized by batch effect 1).

In addition to Nested ComBat, we developed a method for using a Gaussian mixture model (GMM) identifying scan groupings likely split by an unknown covariate based on the observed feature distribution for harmonization (Fig. 6). In this approach, a two-component Gaussian mixture model was fitted for each of the feature distributions. This model can be described by Eq. 1, Where $x$ is a feature with $n$ observations/scans, $i$ is a grouping by an unknown batch effect, and $\phi$ is a coefficient indicating the proportion of the distribution in each sub-distribution (where $\sum\nolimits_{i} {\phi_{i} = 1}$). Any models with less than 25% of the data in either cluster were filtered out, and the model with the highest Akaike Information Criterion (AIC) was automatically selected as the best model to generate the two scan groups for harmonization.

1 $$p\left( x \right) = \mathop \sum \limits_{i = 1}^{2} \phi_{i} N(x | \mu_{i} ,\sigma_{i})$$

After obtaining the scan grouping from using the GMM, ComBat was used to harmonize the whole dataset with the grouping as the batch effect. Whether or not this covariate was an imaging parameter or a clinical covariate of interest is unknown, but in this study, we assumed that the only needed clinical covariates were known and preserved during ComBat harmonization. The harmonized data were then separately harmonized by each of the known batch effects separately with ComBat. The KS test was used to determine the number of features with significant differences in distribution after harmonizing by the grouping alone and harmonizing with the grouping as well as the known batch effects. The code for implementing this algorithm can be found at https://github.com/hannah-horng/generalized-combat.

### Datasets

We used two datasets publicly available from NCI’s The Cancer Imaging Archive (TCIA) to evaluate the performance of our harmonization methods (Table 4)^13^. The first is the Lung3 dataset a set of 86 cases of lung CT scans collected for the study of non-small cell lung cancer (NSCLC)^14^. Of these cases, two cases were dropped from the analysis: one because it was missing clinical covariates, and the other because it was the only case with General Electric as the manufacturer. The second is the NSCLC Radiogenomics dataset collected by researchers at Stanford University, a set of 207 cases of lung CT scans also collected for the study of NSCLC that we will refer to as the Radiogenomics dataset^15^. Of these cases, 12 cases were dropped because the manufacturer was not Siemens or General Electric, and an additional two cases were dropped due to failed feature extraction. Additional acquisition parameters can be found in Table S2, while patient demographics can be found in Table S3. The 3D tumour volume on these images was segmented by a board-certified, fellowship-trained thoracic radiologist with 16 years of clinical experience using the semi-automated ITK-SNAP software (v 3.6.0: http://www.itksnap.org/pmwiki/pmwiki.php?n=Downloads.SNAP3)^16^. Features from lung tumor volumes segmented from both imaging datasets were extracted with the Cancer Imaging Phenomics Toolkit (CapTK) (102 features) and the PyRadiomics software library (430 features), resulting in a total of four sets of features^17,18^. A table of the extracted features can be found in Table S4, 5. In this work, we evaluate two different software packages because while both CaPTk and PyRadiomics are used broadly by the radiomics community and are both IBSI-compliant, they have nuanced differences in their implementation. These differences result in no guarantee of the same feature values obtained from both software and are considered in our analysis^19^.

**Table 4:** Case counts by batch effect for the Lung3 and Radiogenomics datasets.

	Lung3	Radiogenomics
Non contrast-enhanced	34	102
Contrast-enhanced	50	91

	Lung3	Radiogenomics
Low spatial resolution	49	91
High spatial resolution	35	102

	Lung3	Radiogenomics
Siemens	37	54
General electric	–	139
Philips	47	–

### ComBat

All ComBat analyses used the *neuroComBat* Python package, which harmonizes data by a single batch effect [10]. Variation attributable to clinical covariates can be preserved by specifying the clinical variables. The performance of this standard out-of-the-box implementation of ComBat was assessed by applying separate harmonization by each of the three batch effects (contrast enhancement, spatial resolution, manufacturer) (Table 4). In the Lung3 dataset, the clinical variables of death event, histology, stage, gender, and survival were protected. In the Radiogenomics dataset, the clinical variables of death event, histology, sex, smoking status, and days were preserved.

### Method evaluation

Principal components analysis was performed to extract ten radiomic principal components (PCs) from the CapTK and PyRadiomics features in the Lung3 and Radiogenomics datasets for all harmonization methods, capturing 85% of the variance from the radiomic features extracted from each package. The total number of predictors in the case of the Lung3 dataset was capped at 5 out of 10 due to the total number of events (45 deaths) and in the case of the PyRadiomics model was capped at 4 out of 10 due to the number of events (40 deaths) based on the statistical rule of thumb of approximately one predictor per 10 events to avoid model overfitting.

For these models, a five-fold cross-validated multivariate Cox proportional hazards model (200 iterations) was used to compute the concordance index (c-statistic), which measures the ability of the models to predict overall survival. In addition to the cross-validated c-statistics, we also built a model on the complete dataset, to evaluate Kaplan–Meier performance in separating participants above versus below the median prognostic score derived from the model. The log-rank test was used to statistically compare the Kaplan–Meier curves. Models included only the imaging features and did not incorporate additional clinical variables.

## Supplementary Information

Supplementary Information.
