# Supplementary material for: Generalized ComBat harmonization methods for radiomic features with multi-modal distributions and multiple batch effects
Source: Sci Rep. 2022 Mar 16;12:4493. doi: 10.1038/s41598-022-08412-9 (PMC8927332; doi:10.1038/s41598-022-08412-9)
Supplement: Supplementary file 1 — Supplementary Information. [file 41598_2022_8412_MOESM1_ESM.docx]

**Generalized ComBat Harmonization Methods for Radiomic Features with Multi-modal Distributions and Multiple Batch Effects**

**Hannah Horng^1^, Apurva Singh^2^, Bardia Yousefi^2^, Eric A. Cohen^2^, Babak Haghighi^2^, Sharyn Katz^2^, Peter B. Noël^3^, Russell T. Shinohara^4*^, Despina Kontos^2*^**

^1^ Department of Bioengineering, University of Pennsylvania, Philadelphia, PA, 19104

^2^ Center for Biomedical Image Computing and Analysis (CBICA), Department of Radiology, University of Pennsylvania, Philadelphia, PA, 19104

^3^ Laboratory for Advanced Computed Tomography Imaging, Department of Radiology, University of Pennsylvania, Philadelphia, PA, 19104

^4^ Penn Statistics in Imaging and Visualization Endeavor (PennSIVE), Department of Biostatistics, Epidemiology, and Informatics, University of Pennsylvania, Philadelphia, PA, 19104

^*^ Co-senior authors and corresponding authors: [Russell.Shinohara@pennmedicine.upenn.edu](mailto:Russell.Shinohara@pennmedicine.upenn.edu), [Despina.Kontos@pennmedicine.upenn.edu](mailto:Despina.Kontos@pennmedicine.upenn.edu)

**SUPPLEMENTARY INFORMATION**

**Lung3/CAPTK**

|  | Dropped  Features | 5-fold CV  c-statistic | 95% CI | Log-rank  p-value |
| --- | --- | --- | --- | --- |
| Original  + DROP | 65 | 0.52 | [0.45,0.59] | 0.032 |
| Nested  + DROP | 44 | 0.63 | [0.58,0.67] | 0.0061 |
| GMM  + DROP | 38 | 0.48 | [0.42,0.55] | 0.061 |

**Lung3/PyRadiomics**

|  | Dropped  Features | 5-fold CV  c-statistic | 95% CI | Log-rank  p-value |
| --- | --- | --- | --- | --- |
| Original  + DROP | 330 | 0.58 | [0.50,0.66] | 0.0048 |
| Nested  + DROP | 157 | 0.64 | [0.58,0.69] | 0.0006 |
| GMM  + DROP | 330 | 0.58 | [0.52,0.63] | 0.037 |

**Radiogenomics/CAPTK**

|  | Dropped  Features | 5-fold CV  c-statistic | 95% CI | Log-rank  p-value |
| --- | --- | --- | --- | --- |
| Original  + DROP | 53 | 0.56 | [0.52,0.63] | 0.02 |
| Nested  + DROP | 52 | 0.63 | [0.56,0.67] | 0.052 |
| GMM  + DROP | 78 | 0.60 | [0.52,0.62] | 0.035 |

**Radiogenomics/PyRadiomics**

|  | Dropped  Features | 5-fold CV  c-statistic | 95% CI | Log-rank  p-value |
| --- | --- | --- | --- | --- |
| Original  + DROP | 330 | 0.62 | [0.55,0.65] | 0.043 |
| Nested  + DROP | 226 | 0.65 | [0.63,0.72] | 0.00082 |
| GMM  + DROP | 291 | 0.63 | [0.56,0.68] | 0.055 |

**Table S1**. C-statistics and 95% confidence intervals (CI) for 5-fold cross-validated Cox proportional hazard models built from harmonized data. DROP indicates that all features with a statistically significant difference in distribution observed with at least one imaging parameter were removed from the dataset.


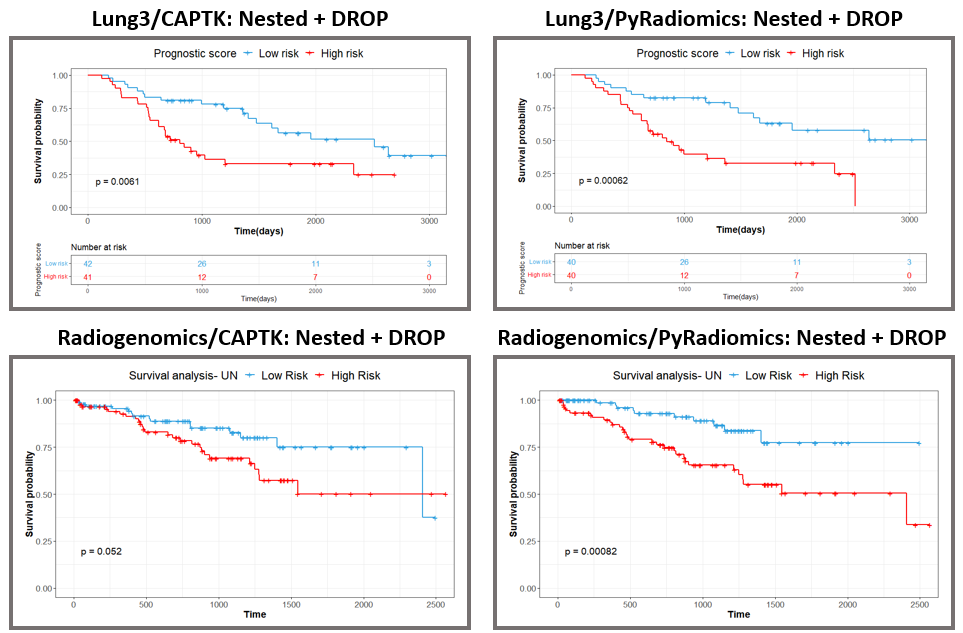


**Figure S1**. In-sample Kaplan-Meier curves fitted on the original features and the harmonization approach with the highest c-statistic.

|  | Lung3 | Radiogenomics |
| --- | --- | --- |
| Peak Kilovoltage | 120-140 kVp | 80-140 kVp |
| Tube Current | 33-463 mA | 100-750 mA |
| Slice Thickness | 1.5-5 mm | 0.625-3 mm |
| Reconstruction Diameter | 308-700 mm | 306-500 mm |
| Reconstruction Kernels | Phillips  A, B, C, D  Siemens  B30f, B30s, B40f, B60f, B70s | General Electric  BONE, BONEPLUS, LUNG, SOFT, STANDARD  Siemens  B19f, B20f, B25f, B31f, B31s, B40f, B45f, B45s, B50f, B60f, B70f |

**Table S2**. CT acquisition parameters for the Lung3 and Radiogenomics datasets.

**Lung3**

| Feature | Subject Counts |
| --- | --- |
| Sex  Male  Female | 57  27 |
| Survival  Yes  No | 39  45 |
| Histology  Adenocarcinoma  Squamous Cell Carcinoma  Other | 40  32  12 |
| Combined Stage  I  II  II  Unknown | 38  25  12  9 |

**Radiogenomics**

| Feature | Subject Counts |
| --- | --- |
| Sex  Male  Female | 128  65 |
| Survival  Yes  No | 152  41 |
| Histology  Adenocarcinoma  Squamous Cell Carcinoma  Other | 159  30  4 |
| Smoking  Nonsmoker  Former  Current | 119  42  32 |

**Table S3**. Patient demographics for the Lung3 and Radiomics datasets.

| Feature Category | Feature Name |
| --- | --- |
| Intensity | *Coefficient of Variation*  *Energy*  *Inter Quartile Range*  *Kurtosis*  *Maximum*  *Mean*  *Mean Absolute Deviation*  *Median*  *Median Absolute Deviation*  *Minimum*  *Mode*  *Ninetieth Percentile*  *Quartile Coefficient of Variation*  *Range*  *Root Mean Square*  *Skewness*  *Standard Deviation*  *Sum Tenth Percentile*  *Variance* |
| Histogram | *Frequency*  *Coefficient of Variation*  *Energy*  *Entropy*  *Fifth Percentile*  *Fifth Percentile Mean*  *Inter Quartile Range*  *Kurtosis*  *Mean*  *Mean Absolute Deviation*  *Median*  *Median Absolute Deviation*  *Mode*  *Ninetieth Percentile*  *Ninety Fifth Percentile*  *Ninety Fifth Percentile Mean*  *Quartile Coefficient of Variation*  *Robust Mean Absolute Deviation*  *Root Mean Square*  *Seventy Fifth Percentile*  *Skewness*  *Standard Deviation*  *Sum*  *Tenth Percentile*  *Twenty Fifth Percentile*  *Uniformity*  *Variance* |
| Volumetric | *Pixels*  *Volume* |
| Morphologic | *Eccentricity*  *Ellipse Diameter*  *Elongation*  *Equivalent Spherical*  *Perimeter*  *Equivalent Spherical Radius*  *Flatness*  *Largest Component Size*  *Number of Pixels*  *Perimeter*  *Physical Size*  *Roundness* |
| Grey level run length matrix (GLRLM) | *Grey Level Non- Uniformity Normalized*  *Grey Level Non-Uniformity*  *Grey Level Variance*  *High Grey Level Run Emphasis*  *Long Run Emphasis*  *Long Run High Grey Level Emphasis*  *Long Run Low Grey Level Emphasis*  *Low Grey Level Run Emphasis*  *Run Entropy*  *Run Length Non- Uniformity Normalized*  *Run Length Non- Uniformity*  *Run Length Variance*  *Run Percentage*  *Short Run Emphasis*  *Short Run High Grey Level Emphasis*  *Short Run Low Grey Level Emphasis*  *Total Runs* |
| Grey level size zone matrix (GLSZM) | *Grey Level Mean*  *Grey Level Non- Uniformity*  *Grey Level Non-Uniformity Normalized*  *Grey Level Variance*  *High Grey Level Emphasis*  *Large Zone Emphasis*  *Large Zone High Grey Level Emphasis*  *Large Zone Low Grey Level Emphasis*  *Low Grey Level Emphasis*  *Small Zone Emphasis*  *Small Zone High Grey Level Emphasis*  *Small Zone Low Grey Level Emphasis*  *Zone Percentage*  *Zone Size Entropy*  *Zone Size Mean*  *Zone Size Non- Uniformity*  *Zone Size Non-Uniformity Normalized*  *Zone Size Variance* |
| Neighboring grey tone difference matrix (NGTDM) | Busyness  *Coarseness*  *Complexity*  *Contrast*  *Strength* |

**Table S4**. Table of CAPTK features used in analysis.

| Feature Category | Feature Name |
| --- | --- |
| Intensity  *All intensity features were also calculated for LoG-sigma 5mm, 4.5mm, 4mm, 3.5mm, 3mm, 2.5mm, 2mm, 1.5mm, 1mm, 0.5mm and for Wavelet LLH, LHL, LHH, HLL, HLH, HHL, HHH, LLL | *Energy*  *Total Energy*  *Entropy*  *Minimum*  *10^th^ percentile*  *90^th^ percentile*  *Maximum*  *Mean*  *Median*  *Interquartile Range*  *Range*  *Mean Absolute Deviation (MAD)*  *Robust Mean Absolute Deviation (rMAD)*  *Root Mean Squared (RMS)*  *Skewness*  *Kurtosis*  *Variance*  *Uniformity* |
| Shape | *Elongation*  *Flatness*  *Least Axis Length*  *Major Axis Length*  *Maximum 2D Diameter-Column*  *Maximum 2D Diameter-Row*  *Maximum 2D Diameter-Slice*  *Maximum 3D Diameter*  *Minor Axis Length*  *Sphericity*  *Surface Area*  *Surface Volume Ratio*  *Volume* |
| Grey level co-occurrence matrix (GLCM) | *Autocorrelation*  *Joint Average*  *Cluster Prominence*  *Cluster Shade*  *Cluster Tendency*  *Contrast*  *Correlation*  *Difference Average*  *Difference Entropy*  *Difference Variance*  *Joint Energy*  *Joint Entropy*  *Informational Measure of Correlation (IMC) 1*  *Informational Measure of Correlation (IMC) 2*  *Inverse Difference Moment (IDM)*  *Maximal Correlation Coefficient (MCC)*  *Inverse Difference Moment Normalized (IDMN)*  *Inverse Difference (ID)*  *Inverse Variance*  *Maximum Probability*  *Sum Entropy*  *Sum of Squares* |
| Grey level size zone matrix (GLSZM) | Small Area Emphasis (SAE)  Large Area Emphasis (LAE)  Grey Level Non-Uniformity (GLN)  Grey Level Non-Uniformity Normalized (GLNN)  Size-Zone Non-Uniformity (SZN)  Size-Zone Non-Uniformity Normalized (SZNN)  Zone Percentage (ZP)  Grey Level Variance (GLV)  Zone Variance (ZV)  Zone Entropy (ZE)  Low Grey Level Zone Emphasis (LGLZE)  High Grey Level Zone Emphasis (HGLZE)  Small Area Low Grey Level Emphasis (SALGLE)  Small Area High Grey Level Emphasis (SAHGLE)  Large Area Low Grey Level Emphasis (LALGLE)  Large Area High Grey Level Emphasis (LAHGLE) |
| Grey level run length matrix (GLRLM) | Short Run Emphasis (SRE)  Long Run Emphasis (LRE)  Grey Level Non-Uniformity (GLN)  Grey Level Non-Uniformity Normalized (GLNN)  Run Length Non-Uniformity (RLN)  Run Length Non-Uniformity Normalized (RLNN)  Run Percentage (RP)  Grey Level Variance (GLV)  Run Variance (RV)  Run Entropy (RE)  Low Grey Level Run Emphasis (LGLRE)  High Grey Level Run Emphasis (HGLRE)  Short Run Low Grey Level Emphasis (SRLGLE)  Short Run High Grey Level Emphasis (SRHGLE)  Long Run Low Grey Level Emphasis (LRLGLE)  Long Run High Grey Level Emphasis (LRHGLE) |
| Neighboring grey tone difference matrix (NGTDM) | Coarseness  Contrast  Contrast 2  Complexity  Strength |
| Grey level dependence matrix (GLDM) | Large Dependence Emphasis (LDE)  Grey Level Non-Uniformity (GLN)  Dependence Non-Uniformity (DN)  Dependence Non-Uniformity Normalized (DNN)  Grey Level Variance (GLV)  Dependence Variance (DV)  Dependence Entropy (DV)  Low Grey Level Emphasis (LGLE)  High Grey Level Emphasis (HGLE)  Small Dependence Low Grey Level Emphasis (SDLGLE)  Small Dependence High Grey Level Emphasis (SDHGLE)  Large Dependence Low Grey Level Emphasis (LDLGLE)  Large Dependence High Grey Level Emphasis (LDHGLE) |

**Table S5**. Table of PyRadiomics features used in analysis.

**Lung3/CAPTK**

|  | Original | ComBat | NestedD | Combo |  |
| --- | --- | --- | --- | --- | --- |
| CE | 10% | 16% | 3% | 5% |  |
| Spatial Resolution | 18% | 21% | 19% | 10% |  |
| Manufacturer | 48% | 45% | 28% | 24% |  |
| Total | 76% | 82% | 50% | 39% |  |

**Lung3/PyRadiomics**

|  | Original | ComBat | NestedD | Combo |  |
| --- | --- | --- | --- | --- | --- |
| CE | 40% | 11% | 2% | 9% |  |
| Spatial Resolution | 43% | 25% | 11% | 19% |  |
| Manufacturer | 61% | 28% | 12% | 24% |  |
| Total | 144% | 64% | 25% | 52% |  |

**Radiogenomics/CAPTK**

|  | Original | ComBat | NestedD | Combo |  |
| --- | --- | --- | --- | --- | --- |
| CE | 17% | 42% | 14% | 12% |  |
| Spatial Resolution | 42% | 43% | 11% | 27% |  |
| Manufacturer | 20% | 51% | 15% | 11% |  |
| Total | 79% | 136% | 40% | 50% | **Total** |

**Radiogenomics/PyRadiomics**

|  | Original | ComBat | NestedD | Combo |  |
| --- | --- | --- | --- | --- | --- |
| CE | 54% | 27% | 9% | 8% |  |
| Spatial Resolution | 69% | 29% | 13% | 27% |  |
| Manufacturer | 44% | 36% | 20% | 11% |  |
| Total | 167% | 92% | 42% | 46% |  |

**Table S6.** Percentage of features with significantly different distributions attributable to batch effects in the original features and after applying standard ComBat, NestedD (dropping with every iteration) ComBat, and standard ComBat harmonization by a variable indicating combination of contrast enhancement, spatial resolution, and manufacturer. Tables contain the percentage of features out of the original number of features with detected significant (p < 0.05) differences in distribution for all batch effects.
